# ChatGPT (GPT-4V) Performance on the Healthcare Information Technologist Examination in Japan

**DOI:** 10.7759/cureus.76775

**Published:** 2025-01-01

**Authors:** Kai Ishida, Eisuke Hanada

**Affiliations:** 1 Faculty of Engineering, Shonan Institute of Technology, Fujisawa, JPN; 2 Faculty of Science and Engineering, Saga University, Saga, JPN

**Keywords:** artificial intelligence, chatgpt, healthcare information technologist, medical education, multimodal large language models

## Abstract

Introduction

The Chat Generative Pretrained Transformer (ChatGPT) has developed rapidly and is used in many fields, including healthcare informatics. This study evaluated ChatGPT (GPT-4V)'s performance on the Healthcare Information Technologist (HCIT) certification exam in Japan, which assesses certified professionals who work with electronic health records to improve patient care.

Methodology

Four hundred seventy-six questions from the HCIT exam were targeted over three years. ChatGPT (GPT-4V) was tested on its ability to answer questions from an HCIT exam to determine if it could perform as well as or better than aspirants taking the exam. Moreover, its performance was evaluated for each academic category, format, presence or absence of images, and calculations.

Results

The mean correct answer rate for all questions was 84%. ChatGPT achieved the exam passing criteria. The correct answer rate for simple-choice (A-type) questions was higher than that for multiple-choice (X2-type) questions (*P* < 0.05). The success rate for questions with images was lower than for text-only questions (*P* < 0.01), and the success rate for questions requiring calculations was lower than for those without calculations (*P* < 0.05).

Conclusions

ChatGPT (GPT-4V) met the passing criteria for the 19th to 21st HCIT exams, suggesting that its performance is effective in passing the HCIT exam. ChatGPT may possess the minimum required knowledge, understanding, and application skills for the HCIT certification.

## Introduction

Artificial intelligence (AI) has quickly gained popularity in various fields. Huge tasks previously performed only by humans are now easily performed by AI-assisted software and robots. In particular, multimodal large language models (MLLMs) can provide answers to questions and also generate new sentences, images, music, and videos. The Chat Generative Pretrained Transformer (ChatGPT), released by Open AI in November 2022, is an MLLM that has attracted attention because of its ability to generate detailed answers to questions in various fields [1].

ChatGPT answers medical questions with a certain degree of accuracy [2,3] and can be applied to various medical areas. For example, it is used to support the diagnosis of common complaints, cancer screening, automatic generation of diagnostic reports, and medical education applications [4-7]. Therefore, ChatGPT can assist medical and healthcare students and professionals. Its ability to pass various medical and healthcare licensing exams has been reported. Many researchers have found that ChatGPT can pass national exams for physicians, pharmacists, nurses, and other healthcare professionals [8-20]. Thus, ChatGPT has some clinical, basic, and related medical knowledge, including pharmacy, diagnostics, rehabilitation, and nutrition. However, we have reported the weaknesses of ChatGPT; for example, ChatGPT could not recognize images and lacked knowledge of standards and medical laws [21].

Healthcare information systems such as electronic medical records and ordering systems have been introduced and used in clinical settings to promote efficiency, reliability, and safety. However, persons with appropriate knowledge are needed to use these systems safely and effectively. A healthcare information technologist (HCIT) is an expert in these systems. Although studies have evaluated the accuracy of purely medical qualification examinations, such as those for physicians, pharmacists, and nurses, studies that examine the performance of MLLMs on questions involving healthcare information technology expertise or knowledge are lacking. This study aimed to evaluate the accuracy of the responses of the current ChatGPT to the HCIT exams in Japan to address this research gap.

## Materials and methods

Overview of HCITs in Japan

An HCIT certified by the Japan Association for Medical Informatics (JAMI) is a professional in the health, medical, and welfare field who can safely and appropriately manage, use, and provide healthcare information based on the characteristics of medical care and using optimal information processing technology to improve the quality and safety of health, medical care, and welfare. The abilities required for an HCIT are categorized into three knowledge and skill areas: medicine and medical care, information processing technology, and healthcare information systems. Additionally, three key abilities are emphasized: communication with other professionals, collaboration with various professions, and coordination to make adjustments between departments and professions.

The HCIT exam assesses whether a candidate has the necessary knowledge and skills to use information processing technology to improve business operations safely, appropriately manage healthcare information, and use medical data. This exam has 160 questions in three sections: healthcare (50 items), information technology (50 items), and health information systems (60 items). Each section also includes 8-10 subsections. Table 1 presents examples of the main topics of each section. Healthcare section contains basic and clinical medicine, diagnosis and treatment, medical records, and clinical databases. Information technology contains hard and software, network, database, and security. Health information systems contain the introduction and operation of systems, standardization, and related law and guidelines. The exam has A-type questions, where an examinee selects one correct answer from five choices and X2-type questions, where two correct answers are selected from five choices. The X2-type will be asked in the healthcare and health information systems sections. There are no specific qualifications to take the exam to become a HCIT. Many of the test takers are healthcare professionals such as nurses, pharmacists, and clinical radiologists, engineers and sales staff from manufacturers that deal with health information systems, and students studying healthcare information. The HCIT exam is held every August with a passing score of approximately 60% or higher for all areas. Approximately 3,000 people take it with a pass rate of about 30%.

**Table 1:** Overview of the main topics of the HCIT exam in each academic field. HCIT, healthcare information technologist; DICOM, Digital Imaging and Communications in Medicine; HL7, Health Level 7; IHE, Integrating the Healthcare Enterprise

	Sub-sections	Main topics
Healthcare	Medicine/Medical general theory	Medical policy and Medical ethics
	Healthcare system	Medical-related laws, Medical professionals, and Regional medical cooperation
	Medical management	Hospital management and Medical safety
	Medical process	Clinical process and Clinical guideline
	Medicine / Pharmacy / Nursing	Basic and clinical medicine, Pharmaceuticals, Clinical nursing
	Diagnosis	Clinical examination and Image diagnosis
	Treatment	Treatment method and Rehabilitation
	Medical records	Various medical records such as initial and progress records, nursing records, and treatment records
	Medical research	Research guidelines, ethics, and evidence
	Medical statistics	Basic statistics and Statistical test
	Clinical database	National database and Cancer registration
Information technology	Information representation	Binary, Hexadecimal, Logical operation, and Data format
	Hardware	Arithmetic logic unit, Control unit, Computer data storage, Input device, and Output device
	Software	Operating system, Application software, and Data algorithms
	Database	Relational database and Data management
	Network	Communication protocol and Network services
	Security	Security management and Encryption technology
	System development	Development process and Project management
	System operation and management	System multiplexing and User management
	Latest technology	Internet of things, Virtual reality, and Agumented reality
Health information system	Characteristics of medical information system	Primary use/secondary use of information, Ethics and responsibilities of information users
	Functions of hospital information system	Electronic medical record, Ordering system, and Other hospital information system
	Introductions of hospital information system	Procurement, Contract, and Requirements
	Operations of hospital information system	Organizational structure and User management
	Healthcare supporting system	Regional medical cooperation system and Telemedicine system
	Medical information standards	Standard master, DICOM, HL7, and IHE
	Law and related guidelines	Personal information protection law and other related laws and guidelines
	Data analysis of medical information	Data analysis of quality of medical care, medical management, and clinical research

Input to the ChatGPT

We targeted 476 questions from the 19th to the 21st HCIT exams held between 2021 to 2023. Four questions were excluded from the scoring. This includes multiple-answer questions with due to infelicity. Table 2 presents the number of questions of the 19th to 21th HCIT exam in each format, excluding 4 infelicity questions. First, we input the sentence “You are aiming to become a Japanese HCIT. Here, you undergo an HCIT exam. Please explain me the correct answer.” into ChatGPT (GPT-4V). We input the Japanese version of the question texts, including images (figures/tables), and options from the HCIT exams for each question. ChatGPT generated answers along with explanations, and we collected and evaluated the content. We compared the answer options provided by ChatGPT with the correct answers from the JAMI website. For the X2-type questions, the correct answer was when both choices were correct. A researcher who has been licensed as HCIT for over 10 years performed the work. The input was performed between March 5 and April 8, 2024.

**Table 2:** Number of questions of the 19th to 21st HCIT exams for each question format. HCIT, healthcare information technologist

Section	Question format	Number of questions
Healthcare	A-type	120
	X2-type	30
Information technology	Questions with images	20
	Text-only question	129
	Questions with calculations	28
	Non-calculation questions	121
Health information technology	A-type	148
	X2-type	29

Analysis

This study calculated the percentage of correct answers for each section. Furthermore, we calculated the correct answer rate for A-type and X2-type questions in the healthcare and health information system sections. We also calculated the percentage of correct answers and the presence or absence of calculations in the information technology sections based on the questions with images and text-only. The statistical analysis of A- and X2-type questions was performed via Microsoft Excel 2021 using the chi-squared test. This test was also used for questions with images, text-only questions and with/without calculations. The significance level was a *P*-value of less than 5%.

## Results

**Figure 1:**
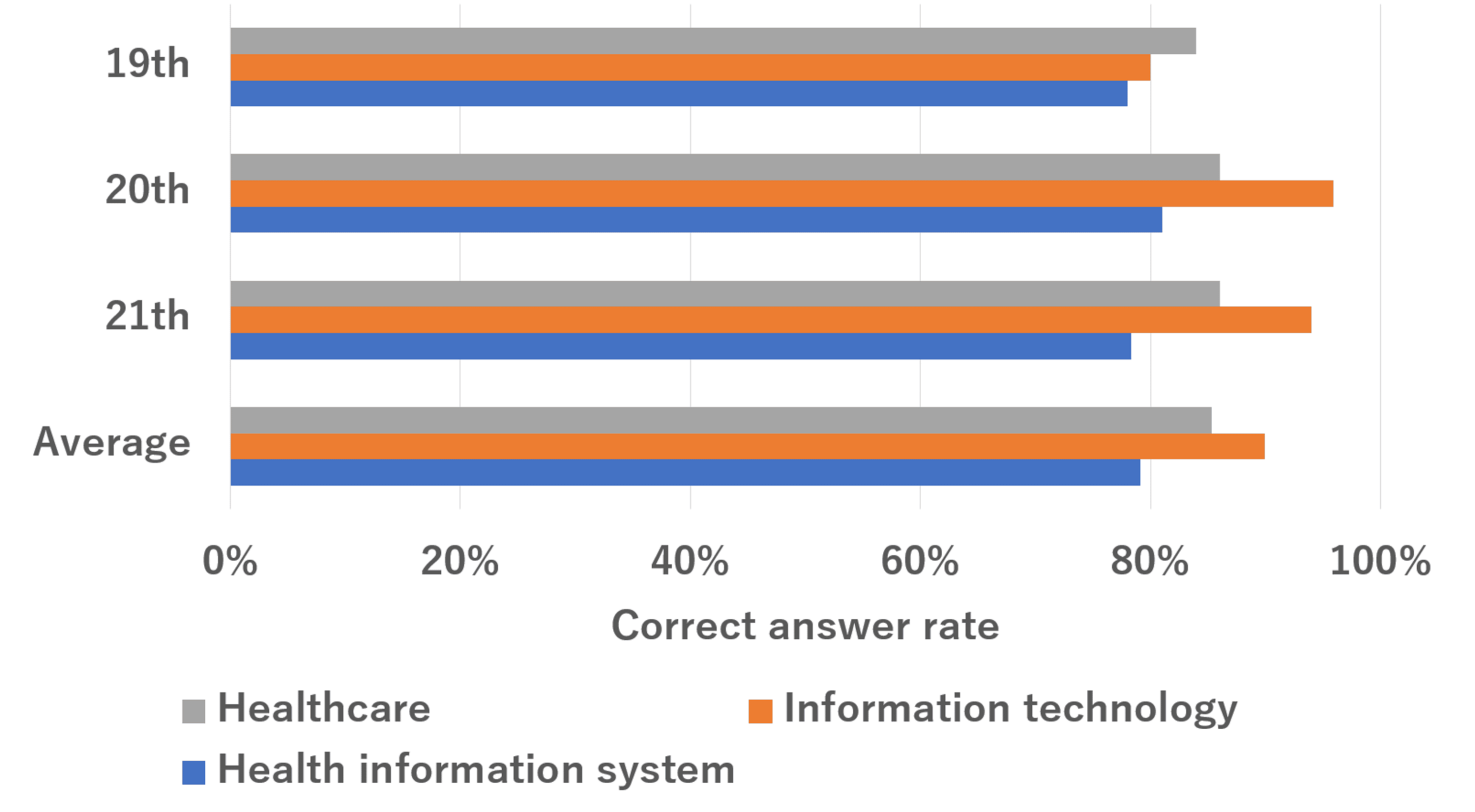
Total ChatGPT (GPT-4V) performance on the 19th-21st HCIT exams. HCIT, healthcare information technologist

**Figure 2:**
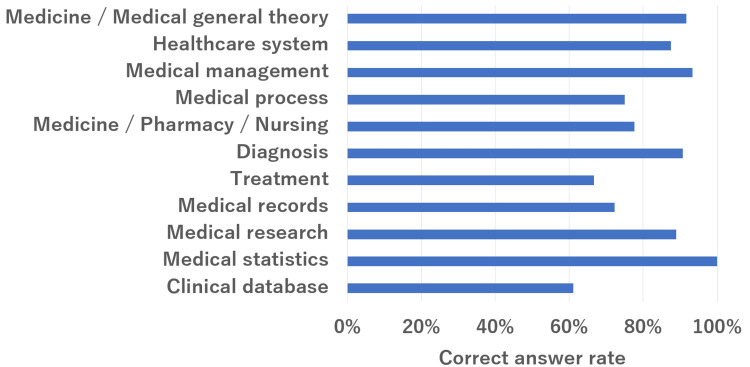
ChatGPT (GPT-4V) performance on the 19th-21st HCIT exam in the healthcare section. HCIT, healthcare information technologist

**Figure 3:**
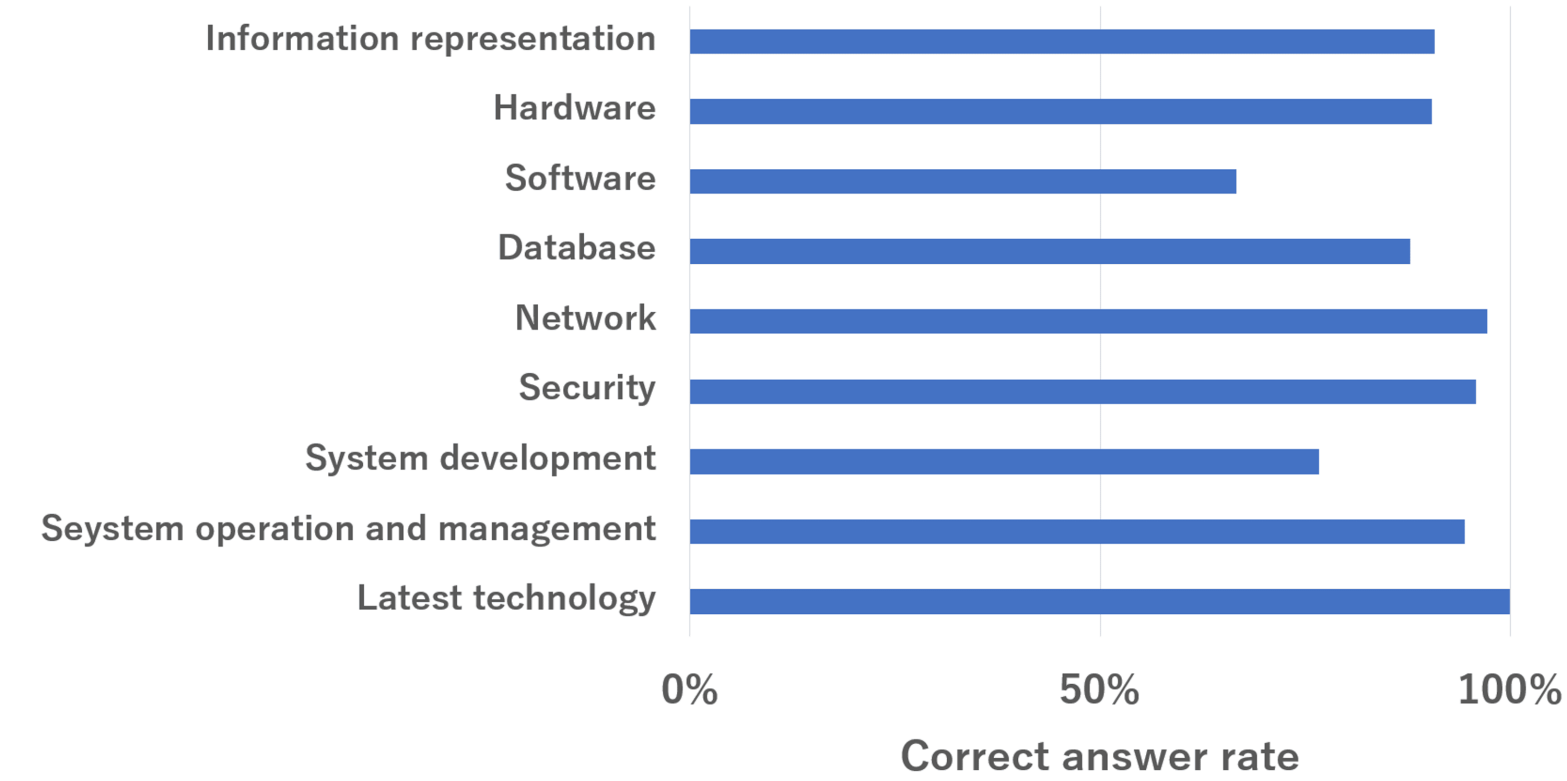
ChatGPT (GPT-4V) performance on the 19th-21st HCIT exam in the information technology section. HCIT, healthcare information technologist

**Figure 4:**
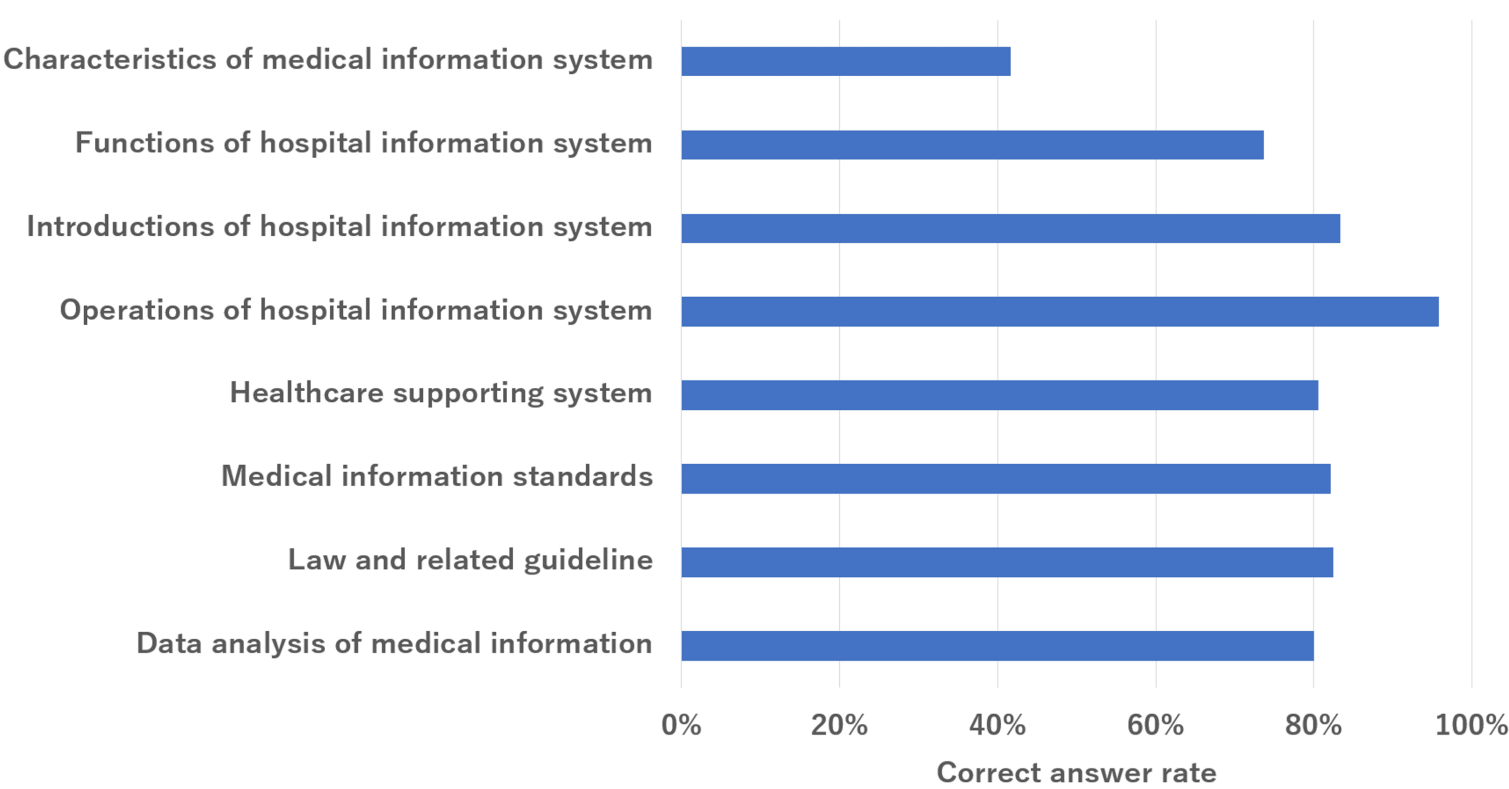
ChatGPT (GPT-4V) performance on the 19th to 21st HCIT exams in health information systems. HCIT, healthcare information technologist

The overall results are summarized in Figure 1. The mean correct answer rate for all questions was 84%. The mean percentages of correct responses for healthcare, information technology, and health information systems were 85%, 90%, and 79%, respectively. The subsection rates for each section are shown in Figures 2-4. In the healthcare section, the mean correct answer rate reached 100% for medical statistics. In the medicine/medical general theory sections, medical management and diagnosis were high. The correct answer was over 90%. Conversely, the treatment and clinical databases did not have a high correct answer rate. It was less than 70%. In the information technology section, the mean correct answer rate reached 100% for the latest technology and close to 100% for network and security. However, software and system development did not have a high correct answer rate. It was less than 80%. In health information systems, the mean correct answer rate was close to 100% for operations of the hospital information system. Conversely, the characteristics of medical systems were low (42%).

**Figure 5:**
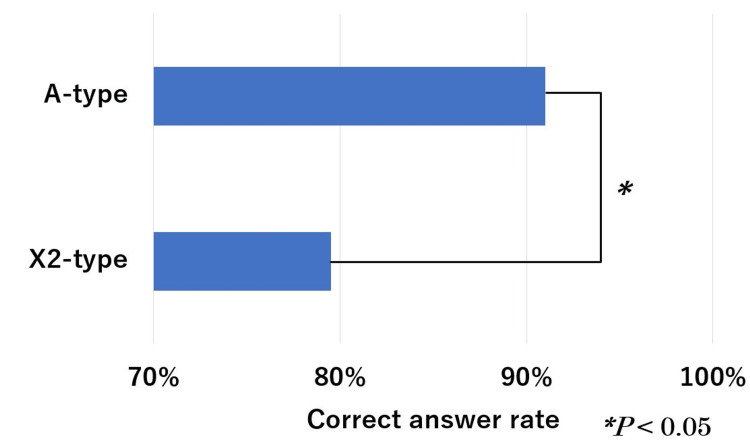
ChatGPT (GPT-4V) performance on the 19th to 21st HCIT exams in question format. HCIT, healthcare information technologist

**Figure 6:**
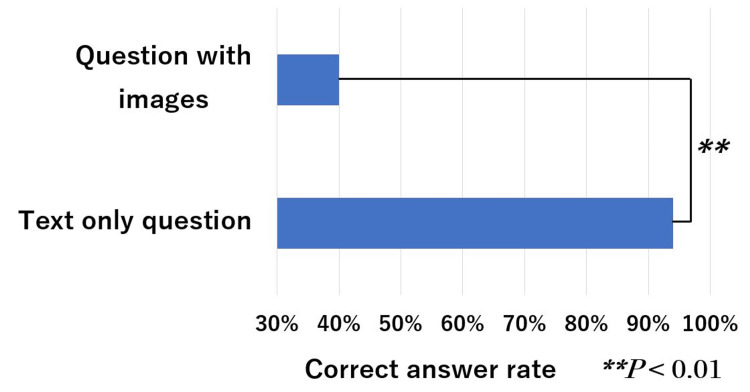
ChatGPT (GPT-4V) performance on the 19th–21st HCIT exams in sections with questions that include images and text-only content. HCIT, healthcare information technologist

**Figure 7:**
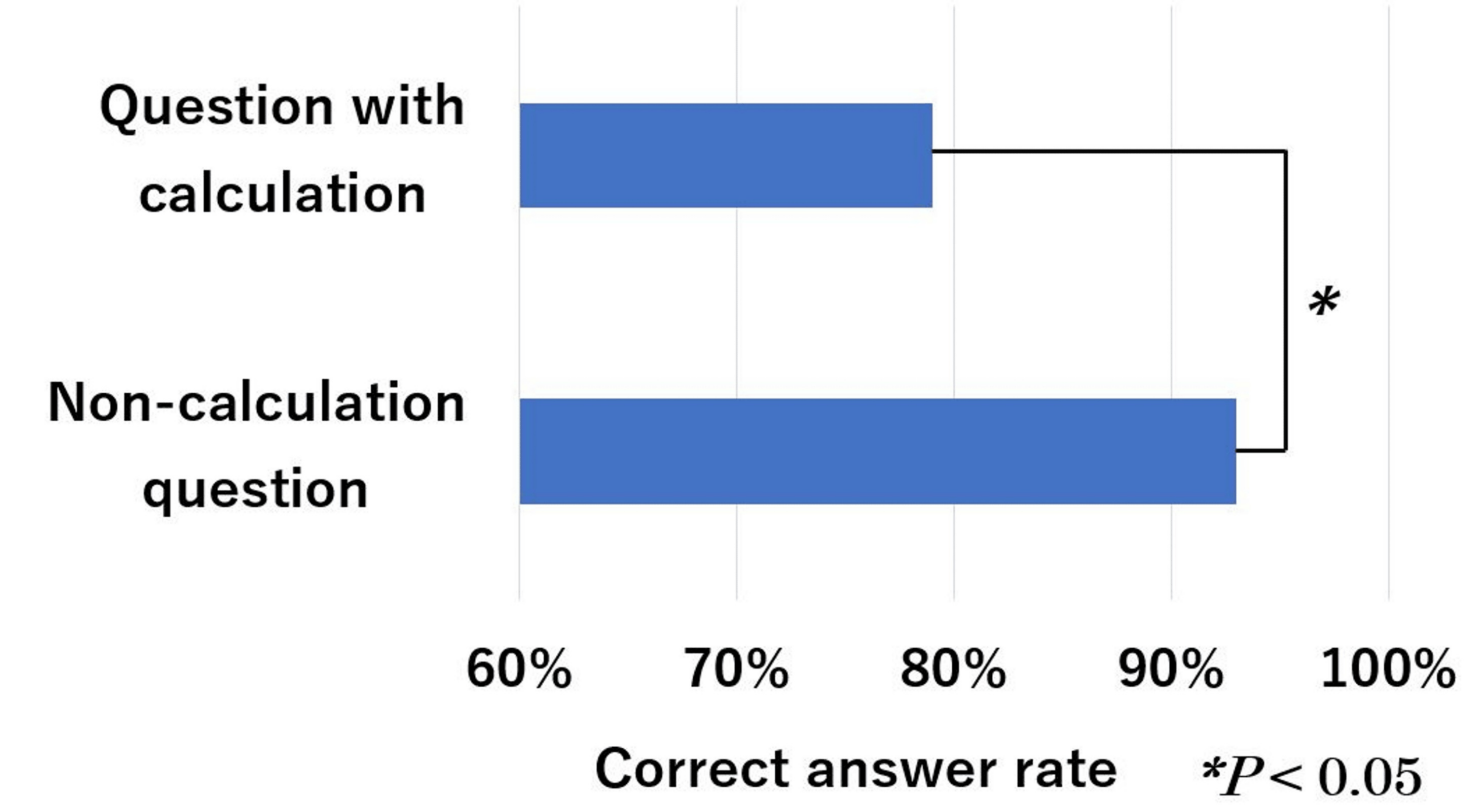
ChatGPT (GPT-4V) performance on the 19th-21st HCIT exams in sections with questions that include images and text-only content. HCIT, healthcare information technologist

The mean correct answer rates for question type, questions with images, text-only questions, and with/without calculations are shown in Figures 5-7. These rates for the A-type and X2-type questions were 91% and 80%, respectively, with a significant difference (*P* < 0.05). These rates for questions with images and text only are 40% and 94%, respectively, with a significant difference (*P* < 0.01). The calculation and non-calculation question rates were 79% and 93%, respectively, with a significant difference (*P* < 0.05).

**Figure 8:**
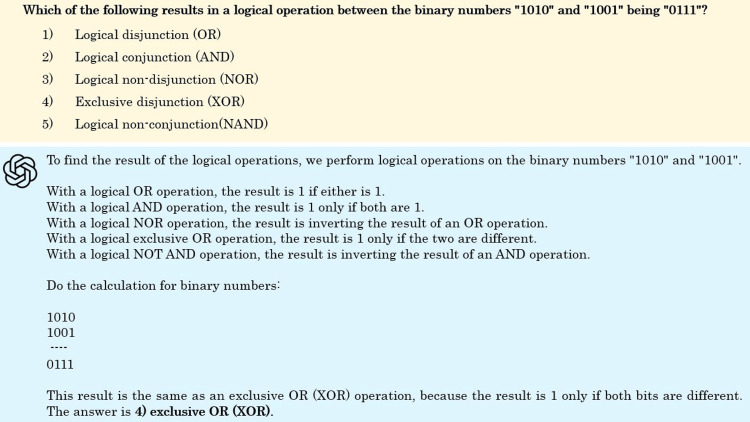
Sample question for an incorrect answer in the information technology section.

**Figure 9:**
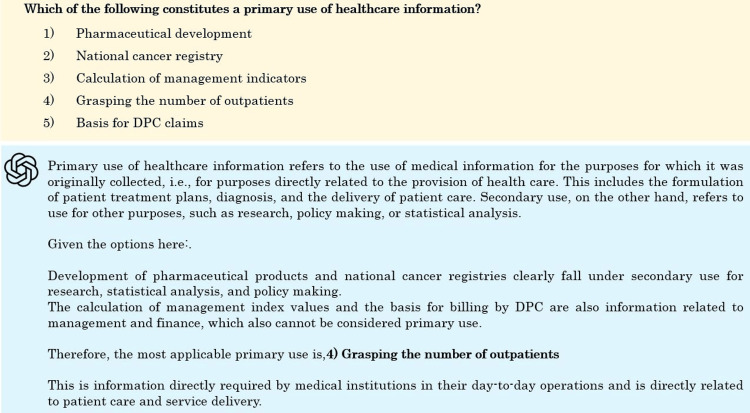
Sample question for an incorrect answer in the health information systems section.

Here are some incorrect questions. Figure 8 is a question in the information technology section, focusing on logical operations. While the explanation of each operator is correct, the correct calculation is not performed, making the answer incorrect. The correct answer is 5) Logical Non-Conjunction (NAND). Figure 9 is a question about the characteristics of medical information systems, from the health information systems section. The question asks about the primary use of healthcare information. While the explanations of primary use and secondary use are correct, the explanation for option 4) is incorrect. Additionally, the correct answer is listed as 5) secondary use, but the correct answer should be primary use.

## Discussion

Our results show that the correct answer rate by ChatGPT for all sections reached more than 75%. Thus, ChatGPT (GPT-4V) meets the passing criteria for the HCIT exams. This suggests that ChatGPT exhibits the performance required to pass the HCIT exam. This multiple-choice question exam assesses not only memory but also understanding, application, analysis, and evaluation skills [22,23]. Therefore, ChatGPT may possess the minimum required knowledge, understanding, and application skills necessary for an HCIT.

According to our results, the percentage of correct answers was high for basic/clinical medicine, diagnostics, networks, and security, which require textbook-based knowledge. It is possible that the high rate of correct answers was due to the large amount of learning resources available on the World Wide Web. Alternatively, for some categories, not only knowledge from textbooks but also knowledge from practical experience may be advantageous. For instance, practical knowledge is necessary in categories such as the characteristics of medical information and development, functionality, and implementation of medical information systems. Compared to healthcare and information technology, there is less learning data on the web, and it is thought that the accuracy was low due to the lack of learning in ChatGPT. Additionally, laws, guidelines, standards, etc. are revised and change every certain number of years. Therefore, since knowledge from the past (before the revision) and present (after the revision) is mixed, it is conceivable that there is a high possibility of incorrect answers. It has been reported that the accuracy of answers regarding laws and related standards in other qualification exams is worse than in multiple fields [21].

In this study, there was a significant difference between the correct answer rate for A- and X2-type questions. However, there is no significant difference between them in Japanese physical therapist national exams [19]. It is thought that the level of difficulty depends on the question format, which varies depending on the field in question and the type of qualification. Correct answer rates were low for questions that required image recognition and calculations. According to past research, the correct answer rate on ChatGPT generally decreases in questions that include images, tables, or diagrams [20]. Questions that require calculations would be worse than simple questions that do not require calculations [21]. Specifically, the correct answer rate was low for questions that required recognizing images, creating calculation formulas, and substituting numbers. At present, the accuracy of recognizing images is currently an issue. If precision were improved, the results would be better.

This study has limitations. First, the ChatGPT output changes depending on the content of the input prompt [24]. In the study targeting ChatGPT (GPT-4) for the Japanese national medical licensing exam, this tool successfully met the minimum passing threshold by adjusting the prompt using the previous year’s questions and performing input after tuning, such as translating from Japanese to English, [13]. Several studies on ChatGPT (GPT-4) that targeted the Japanese national medical and healthcare licensing examinations only considered simple prompts [12-14, 17-19, 21]. However, almost all studies met the passing threshold even without special prompt adjustments or translation from Japanese to English. The ChatGPT-4 performance considerably improved compared with the previous version (GPT-3.5). Even without special prompt engineering, ChatGPT-4 can pass national medical and healthcare examinations. OpenAI reported a slight difference in performance related to language differences in GPT-4 [25]. Therefore, we implemented only one type of prompt. Other special prompt engineering was not considered. Thus, the results may cause changes per question. On the other hand, since the accuracy of answers to questions in the same field was roughly the same, we believe that we have been able to make a certain level of evaluation based on the accuracy of answers provided by ChatGPT. Among the many MLLMs, we only targeted ChatGPT (GPT-4V). For this reason, we targeted ChatGPT as it has received relatively high attention in the field of MLLM, where research progresses extremely rapidly. Previous research on answer analysis for medical and healthcare licensing examinations was based on ChatGPT [7-21]. In the future, it may be possible to obtain more accurate results by targeting other MLLMs or a newly emerging one. Moreover, HCIT qualification in Japan is not universal to other countries. In interpreting the results, it needs to be compared to an equivalent professional exam. However, we found no studies that evaluated the accuracy of ChatGPT for other equivalent healthcare information qualification exams. Additionally, the HCIT exam in Japan consists solely of multiple-choice questions and does not include other formats, such as descriptive or argumentative questions. Therefore, this exam may not fully assess ChatGPT's overall performance. In addition, the study does not provide a detailed analysis of the questions answered incorrectly. If incorrect answers are due to gaps in ChatGPT's knowledge, the patterns of incorrect responses might differ across various fields.

Finally, based on the results so far, we will discuss prospects. ChatGPT's answers to the HCIT exam were highly accurate, and many of the explanations for the answers were appropriate, so we believe it can contribute to the learning of those aiming to take this exam. In addition, in this evaluation, the accuracy of the answers to the healthcare information systems section was low, but if the accuracy of the answers increases with future improvements to the MLLM, it may be possible to contribute to supporting system implementation and operation. However, in all use cases, it is desirable to utilize the generated content while carefully examining it.

## Conclusions

This study evaluated the performance of ChatGPT (GPT-4V) in the Japanese HCIT exam using 476 questions from 2021 to 2023. The mean correct answer rate for all questions was 84%. Although GPT-4V performed considerably well in some academic fields, we found that its correct answer rate was lower for questions based on practical experience, knowledge of standards and regulations, as well as those involving figures/tables and calculations. This rate was low. Although general medical knowledge and some information technology knowledge appear sufficient, the correct answer rate could improve with updates to existing and new models.
